# Immunotherapy targeting liver cancer tumor-initiating cells: challenges, mechanisms, and emerging therapeutic horizons

**DOI:** 10.3389/fimmu.2025.1621243

**Published:** 2025-06-11

**Authors:** Yinying Chai, Tinghui Xu, Xin Chen, Bihua Chen, Xinghai Du, Zhezhong Zhang

**Affiliations:** ^1^ The First Affiliated Hospital of Zhejiang Chinese Medical University (Zhejiang Provincial Hospital of Chinese Medicine), Hangzhou, Zhejiang, China; ^2^ State Key Laboratory of Systems Medicine for Cancer, Shanghai Cancer Institute, Renji Hospital, Shanghai Jiao Tong University School of Medicine, Shanghai, China

**Keywords:** tumor-initiating cells, liver cancer, tumor microenvironment, therapeutic resistance, immunotherapy, nanotechnology, genome editing, non-coding RNAs

## Abstract

Liver cancer is a major global health burden, with hepatocellular carcinoma (HCC) being the most common type. Liver cancer tumor-initiating cells (TICs) are responsible for recurrence, metastasis, and therapeutic resistance, thereby presenting formidable treatment challenges. This review provides a comprehensive summary of the biological features of liver cancer TICs, including their potential cellular origins, diagnostic difficulties, key signaling pathways, and complex interactions with the tumor immune microenvironment. Special emphasis is placed on immunotherapeutic strategies, which have shown notable progress but remain limited by TIC-induced immune resistance. The review discusses current approaches such as immune checkpoint inhibitors (ICIs), adoptive cell therapies, and tumor vaccines, as well as combination strategies integrating immunotherapy with chemotherapy, targeted therapy, and locoregional interventions. Furthermore, emerging strategies including gene editing, targeted tyrosine kinase inhibition, and artificial intelligence-based tumor prediction are being explored for their potential to improve therapeutic efficacy. The significance of this review lies in highlighting the importance of surmounting the challenges presented by TICs to boost the efficacy of liver cancer treatment. In conclusion, although existing treatment approaches have demonstrated promise, further research is warranted to elucidate the origins of TICs, establish accurate diagnostic methods, and overcome resistance, ultimately enhancing the efficacy of liver cancer treatment and improving patient outcomes.

## 1 Introduction

Liver cancer poses a significant global health burden, ranking as the sixth most common cancer and the fourth leading cause of cancer-related deaths worldwide, with hepatocellular carcinoma (HCC) accounting for approximately 90% of cases (1, 2). Despite advancements in diagnosis and treatment, the five-year survival rate for liver cancer remains suboptimal, primarily due to high rates of recurrence, metastasis, and therapeutic resistance (3–5).

Tumor-initiating cells (TICs) in liver cancer, a subpopulation within tumors, contribute to these therapeutic challenges owing to their self-renewal capacity, high plasticity, and tumorigenic potential (6, 7). These cells exhibit distinct molecular signatures, including the expression of surface markers such as CD133 and EpCAM, and they mediate dysregulated signaling pathways such as Wnt/β-catenin and Sonic Hedgehog (SHH) (8–10). Furthermore, interactions between liver cancer TICs and the tumor microenvironment (TME)—particularly through metabolic crosstalk with cancer-associated fibroblasts (CAFs) and immunosuppressive functions mediated by immune cells—establish a complex niche that significantly complicates therapeutic interventions (11, 12).

Immunotherapy is a breakthrough treatment for Liver cancer TICs. However, the heterogeneity and adaptive resistance mechanisms of Liver cancer TICs limit their efficacy (13, 14). To overcome these obstacles, current therapeutic strategies employ combination therapies, such as atezolizumab and bevacizumab, to enhance treatment response rates (15). Despite progress, debates continue regarding the cellular origins of liver cancer TICs and their role in tumor heterogeneity, highlighting the need for further research to better understand their biological and clinical significance (16).

This review explores the role of TICs in liver cancer, current diagnostic techniques, and therapeutic strategies, while also discussing emerging technologies and future perspectives, particularly in immunotherapy, to enhance liver cancer treatment.

## 2 Controversies and challenges in liver cancer TICs research

### 2.1 Contested origins of liver cancer TICs

The origin of liver cancer TICs remains debated. One prominent hypothesis suggests that TICs may stem from hepatic progenitor cells (HPCs). Specifically, CD34+ HPCs, essential for liver regeneration, have been proposed as a potential source of liver cancer stem cells (LCSCs). In a study by Park et al., CD34+ cells isolated from the PLC/PRF/5 hepatoma cell line were found to function as TICs, capable of generating HCC, cholangiocarcinoma (CC), and combined hepatocellular cholangiocarcinoma (CHC) in immunodeficient mice (17). These cells expressed both hepatic and hematopoietic markers, suggesting they might result from the fusion of hepatobiliary stem cells and myeloid precursors. This fusion likely provides tumor cells with cross-lineage characteristics, which contribute to tumor heterogeneity and malignant progression (18). Moreover, subpopulations of TICs that express specific surface antigens like CD133, CD44, and CD90 show distinct tumorigenic potentials, further contributing to the histological diversity observed in liver cancers (17).

An alternative theory involves the plasticity of mature hepatocytes. Evidence indicates that mature hepatocytes can serve as the origin of intrahepatic cholangiocarcinoma (ICC) cells. Mesencephalic astrocyte-derived neurotrophic factor (MANF), upregulated in ICC, promotes the transformation of mature hepatocytes into ICC cells via its interaction with CK19 and activation of the Notch signaling pathway (19). Furthermore, hepatocytes have the potential to transform directly into cancer cells following sequential genomic damage and dedifferentiate into precursor cells expressing progenitor cell markers (20).

### 2.2 Challenges in diagnosing liver cancer TICs

The diagnosis of liver cancer TICs is fraught with challenges, primarily stemming from the lack of specific biomarkers. While markers such as CD133, EpCAM, and certain lncRNAs have been associated with liver TICs, their diagnostic accuracy remains constrained due to non-exclusive expression in other liver or tumor microenvironment cell types, which can lead to false positives or negatives (21). Compounding this issue is the complexity of the TME, where immunosuppressive factors—including immune cell infiltration, stromal components, and secreted molecules—can modify biomarker expression and compromise detection accuracy (22, 23). Additionally, the spatial and temporal heterogeneity of liver tumors, characterized by variations in genetic mutations, epigenetic changes, and cellular composition across different tumor regions, further hinders the development of universal diagnostic methods (24, 25). Clinically, the difficulty of early-stage detection in primary liver cancer, coupled with limited treatment options, exacerbates the challenge of identifying TICs at actionable stages. Classic biomarkers like AFP and des-gamma-carboxy prothrombin (DCP) exhibit low sensitivity and specificity for early detection (26), while newer biomarkers identified through proteomic, metabolomic, and genomic analyses hold promise but require validation for routine clinical use (27).

### 2.3 Resistance mechanisms of liver cancer TICs to therapy

Therapeutic resistance poses a significant obstacle in managing liver TICs. A key example is the response of TICs to sorafenib, a first-line treatment for advanced hepatocellular carcinoma. While sorafenib initially suppresses the proliferation of certain HCC cell lines, it paradoxically causes a transient expansion of TIC populations. These TICs activate the YAP signaling pathway, a potent oncogenic transcriptional coactivator that enhances tumor cell survival, proliferation, and invasion (28). Resistant TICs display increased sphere-forming ability, higher proportions of EpCAM-positive cells, and upregulated YAP expression (29), all of which are associated with stemness and aggressive tumor behavior. Notably, combining sorafenib with a YAP inhibitor has shown synergistic anti-tumor effects, suggesting potential for dual pathway inhibition.

Epigenetic reprogramming also contributes to TIC-mediated resistance. In sorafenib-resistant HCC, EZH2 overexpression enhances NOTCH1 signaling through H3K27me3-mediated chromatin modifications, supporting TIC self-renewal and tumorigenicity (30). Inhibiting EZH2 can restore sorafenib sensitivity by suppressing NOTCH1 and weakening TIC properties, offering a promising strategy to overcome resistance.

## 3 Role and molecular pathways of TICs in liver cancer pathogenesis

### 3.1 Molecular mechanisms underpinning stemness and progression of liver cancer TICs

Liver cancer TICs rely on multiple molecular pathways to sustain their stemness and drive liver Cancer progression. A crucial pathway in this process is the Wnt/β-catenin pathway. For instance, LncTIC1 interacts with the N-terminal of β-catenin, inhibiting its phosphorylation and stabilizing the protein to activate Wnt/β-catenin signaling, thereby promoting TIC self-renewal and tumorigenesis (9). Similarly, TFAP4 amplifies this signaling cascade by directly binding to the promoters of DVL1 and LEF1, further reinforcing TIC characteristics (31). Additionally, activation of the Wnt/β-catenin pathway promotes M2 polarization of tumor-associated macrophages (TAMs), which facilitate immune evasion by secreting immunosuppressive cytokines such as IL-10 and TGF-β (32). Another critical regulator of liver cancer TICs is the interleukin-8 (IL-8) signaling axis. CD133+ liver TICs exhibit significant dysregulation of this pathway, evidenced by genomic and proteomic overexpression of IL-8 (8). This aberrant expression promotes endothelial cell proliferation, tube formation, and TIC stemness maintenance. Mechanistically, NTS binding to NTSR1/2 on CD133+ TICs triggers MAPK phosphorylation, driving IL-8 and CXCL1 production. IL-8 then acts via CXCR1/2 in an autocrine/paracrine manner to sustain MAPK activation, forming a positive feedback loop (8).

**Figure 1:**
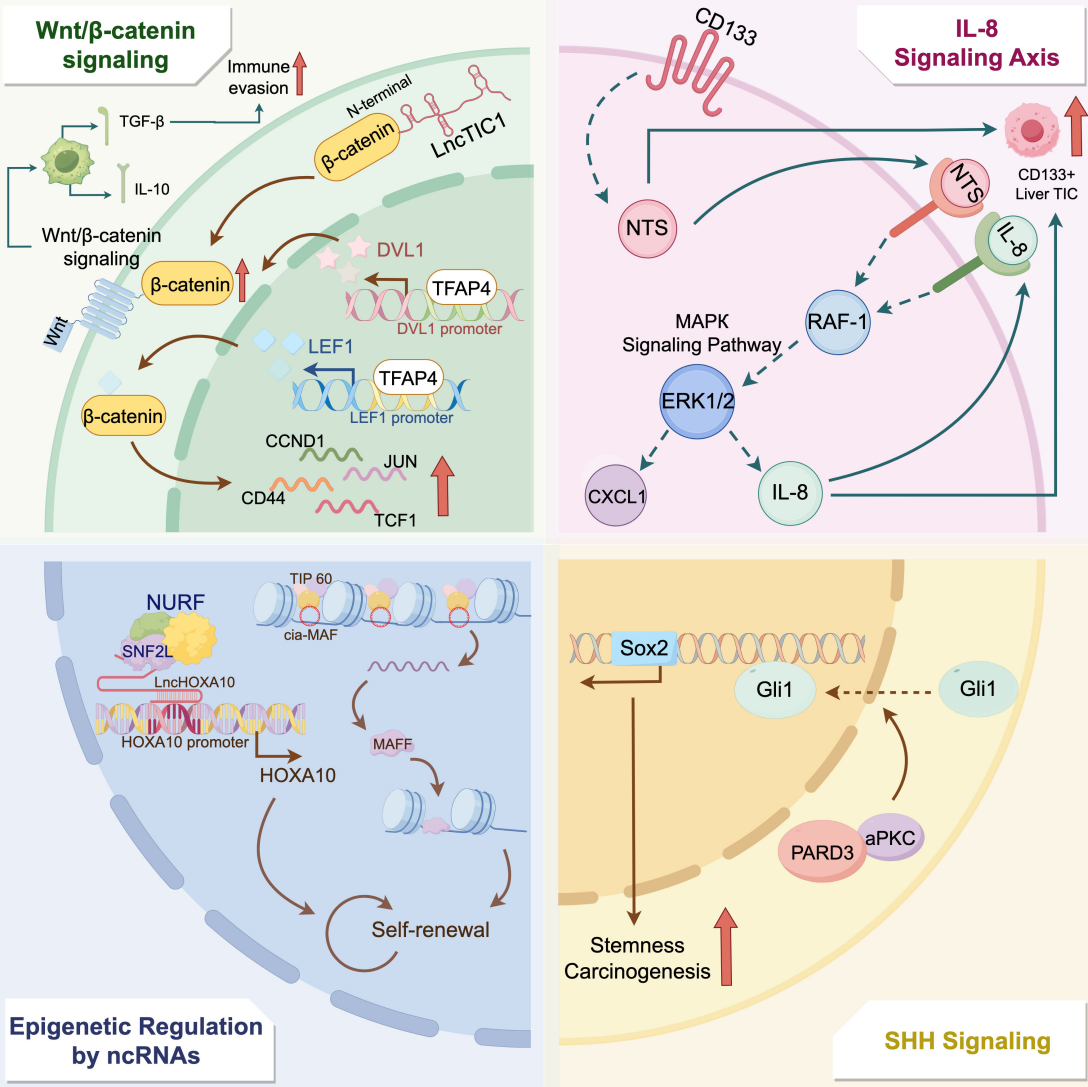
Molecular mechanisms regulating liver cancer tumor-initiating cells stemness and tumor progression. This figure illustrates the intricate molecular pathways that regulate the stemness and tumorigenic potential of liver cancer tumor-initiating cells (TICs). The interaction between LncTIC1 and β-catenin increases the stability of β-catenin, thereby activating the Wnt/β-catenin pathway. TFAP4 binds to the promoters of DVL1 and LEF1, further enhancing β-catenin stability and activating the expression of downstream genes such as CCND1, CD44, JUN, and TCF1. Additionally, this pathway promotes M2 macrophage polarization, facilitating immune evasion through the secretion of IL-10 and TGF-β. The IL-8 signaling axis, dysregulated in CD133+ liver TICs, contributes to TIC stemness by enhancing MAPK signaling and forming a positive feedback loop via IL-8 and CXCL1. Moreover, epigenetic regulation by non-coding RNAs, such as cia-MAF and lncHOXA10, modulates TIC self-renewal. Circular RNA cia-MAF binds to the MAFF promoter and recruits the TIP60 histone acetyltransferase complex, driving MAFF expression, while the long non-coding RNA lncHOXA10 interacts with SNF2L and recruits the NURF chromatin remodeling complex to the HOXA10 promoter in liver cancer TICs. Finally, SHH signaling, mediated by the PARD3-aPKC interaction, contributes to the upregulation of stemness genes such as SOX2, which correlates with increased stemness and carcinogenesis.

Epigenetic regulation by non-coding RNAs further modulates TICs stemness and metastatic potential. For instance, the circular RNA cia-MAF binds to the MAFF promoter and recruits the TIP60 histone acetyltransferase complex, driving MAFF expression which is a transcription factor critical for TIC self-renewal and metastasis. Likewise, the long non-coding RNA lncHOXA10 interacts with SNF2L and recruits the NURF chromatin remodeling complex to the HOXA10 promoter in liver cancer TICs, contributing to HOXA10 expression and liver cancer progression (33). These epigenetic mechanisms also synergize with canonical signaling pathways. For example, SHH signaling, activated by the PARD3-aPKC interaction, upregulates stemness genes such as SOX2 and correlates with advanced tumor stages (10) (**Figure 1**).

### 3.2 TICs-TME crosstalk in liver cancer progression

The TME, which consists of a variety of non-cancerous cells, extracellular matrix components, and soluble factors, plays a critical role in influencing the behavior of liver cancer TICs. A case in point is CAFs, key stromal cells in the TME, interact with TICs to promote tumor progression. In a three-dimensional (3D) co-culture organoid model of primary murine liver tumors, the presence of CAFs significantly increased the number of LGR5+ TICs in the tumor compared to organoids lacking CAFs. This observation suggests that CAFs support LGR5+ liver cancer TICs, facilitating tumor formation, growth, and metastasis (34). Additionally, CAFs can enhance the stemness of TICs by secreting cytokines such as interleukin-6 (IL-6), which activate signaling pathways like IL-6-STAT3-NOTCH, thereby promoting tumor progression (35).

TICs interact with immune cells within the TME to establish an immunosuppressive milieu. Specifically, TREM2 macrophages are associated with glycolysis and PKM2 expression in HCC cells, likely mediated by the secretion of IL-1β, which promotes malignant phenotypes of HCC cells (36). Meanwhile, CD49f-high TICs recruit tumor-promoting neutrophils through the CXCL2-CXCR2 axis, establishing an immunosuppressive environment within the TME (12). This crosstalk between TICs and immune cells highlights the intricate tumor-immune microenvironment and holds important implications for the development of effective immunotherapy strategies (37).

## 4 Therapeutic strategies targeting liver cancer TICs

### 4.1 Immunotherapy approaches for liver cancer TICs

Immunotherapy is a promising therapeutic approach for liver cancer TICs. Among various immunotherapeutic agents, immune checkpoint inhibitors (ICIs) are a prominent class. In the treatment of HCC, the combination of the anti-programmed cell death ligand 1 (PD-L1) antibody atezolizumab and the VEGF-neutralizing antibody bevacizumab has demonstrated significant efficacy. Consequently, this regimen is now recommended as the first-line systemic therapy for advanced HCC (15). Additionally, combining different ICIs, like nivolumab and ipilimumab, has yielded promising outcomes in second-line treatments, as evidenced by the CheckMate 040 study (38). Nevertheless, ICI monotherapy demonstrates limited efficacy in HCC, with objective response rates of 10–20% and no significant overall survival (OS) improvement observed in phase III clinical trials such as CheckMate 459 and KEYNOTE-240 (39).

Adoptive cell therapy (ACT) and peptide- or dendritic cell (DC)-based vaccines are also under active investigation. ACT, including chimeric antigen receptor T-cell (CAR-T) therapy targeting tumor-associated antigens (TAA) like glypican-3 (GPC3) and alpha-fetoprotein (AFP), has demonstrated encouraging results in early-phase clinical trials by specifically recognizing and eliminating tumor cells without MHC restriction (40).

Peptide-based vaccines, exemplified by GPC3-targeted formulations, induce cytotoxic T lymphocyte (CTL) infiltration into tumors and can improve overall survival in HCC patients (41). Similarly, DC vaccines targeting tumor antigens or neoantigens help remodel the immunosuppressive TME, by activating tumor-reactive T cells, showing good safety and immunogenicity profiles in early trials (42, 43). However, the liver’s unique immune microenvironment limits immune cell infiltration and function, reducing the effectiveness of these therapies. Therefore, identifying predictive biomarkers for immunotherapy response remains crucial.

### 4.2 Combination therapies for liver cancer TICs

#### 4.2.1 Synergizing immunotherapy with chemotherapy

Cytotoxic chemotherapy, when combined with ICIs, has demonstrated improved antitumor efficacy against liver cancer TICs. Recent clinical studies have shown that the FOLFOX regimen combined with immunotherapy achieves an objective response rate of approximately 60%, with around 25% of patients successfully converted to surgical candidates (44). Conjugates linking irinotecan with TDO inhibitors demonstrated enhanced cellular uptake and cytotoxicity in TDO-overexpressing HepG2 cells, induced G2 phase arrest and mitochondrial apoptosis, and inhibited kynurenine production and aryl hydrocarbon receptor signaling, thereby promoting T cell activation (45).

#### 4.2.2 Synergizing immunotherapy with targeted agents

The combination of immunotherapy and molecular-targeted therapies is actively being explored. For instance, combining PD-1 blockade with pegylated interferon-α (Peg-IFNα) has been shown to increase cytotoxic CD8+ T-cell infiltration, restore anti-tumor immunity, promote tumor cell apoptosis, and reduce angiogenesis in HCC models (46). Another study by Hsieh et al. demonstrated that the combination of bavituximab and pembrolizumab increased the response rate in HCC patients from 16% to 32%, highlighting the potential of combination therapies (47).

#### 4.2.3 Combining systemic immunotherapy with locoregional therapies

Locoregional therapies combined with systemic immunotherapy represent a promising strategy for targeting liver cancer TICs. One innovative approach, transcatheter arterial chemo-immuno-embolization (TACIE), integrates transcatheter arterial chemoembolization (TACE) with immune adjuvants like TLR9 agonists. This combination has exhibited synergistic antitumor effects in preclinical models by enhancing systemic immunity and promoting tumor regression, while maintaining safety (48). This method has shifted treatment strategies from “total embolization,” aimed at complete tumor necrosis, to “partial embolization,” which reduces tumor burden while preserving liver function. It also provides an immune boost that enhances the effects of immunotherapy and targeted agents (49).

In addition, radiofrequency ablation (RFA) combined with ICIs can boost systemic immune activation via neoantigen release and abscopal effects (50). Stereotactic body radiation therapy (SBRT), in conjunction with ICIs, is also being explored to target oligometastases and improve rates of radical cure (51) (**Table 1**).

**Table 1:** Immunotherapeutic approaches and combinatorial strategies targeting liver cancer tumor-initiating cells.

Therapeutic Strategy	Specific Therapy	Mechanism	Clinical Effect or Research Progress
Immunotherapy	Combination of ICIs	Atezo inhibits PD-L1, bevacizumab Beva neutralizes VEGF, and nivolumab Nivo and Ipi target the PD-1 and CTLA-4 pathways, respectively, to enhance immune system activation	Atezo + Beva is recommended as first-line treatment for advanced HCC, improving survival and quality of life; In the CheckMate 040 second-line treatment, the combination of Nivo + Ipi achieves an ORR of over 30%, with some patients experiencing extended survival.
	ACT	CAR-Tcells are genetically engineered to express receptors that recognize tumor-associated antigens, thereby activating T cells to target and eliminate tumor cells	In early-stage trials, CAR-T therapy targeting GPC3 has shown cases of objective response, with some patients experiencing stable or improved conditions.
	Peptide or DC vaccines	The GPC3-targeted peptide vaccine stimulates the body to produce CTLs against GPC3, which then infiltrate the tumor; DC vaccines uptake and present antigens to activate T cells and remodel the immune microenvironment	The GPC3-targeted peptide vaccine induces a CTL response, leading to decreased tumor markers and extended survival in some patients; DC vaccines demonstrate good safety and some efficacy in early HCC trials.
Combination Therapies	Immunotherapy + Chemotherapy	PD-1 blockade + FOLFOX: PD-1 blockade boosts immune recognition, while FOLFOX induces antigen release via immunogenic cell death TDO inhibitor + irinotecan: the conjugate enters cancer cells to induce cancer cells apoptosis and concurrently promote T-cell proliferation via inhibiting TDO protein expression	PD-1 blockade + FOLFOX achieves an objective response rate of approximately 60%, with around 25% of patients successfully converted to surgical candidates; Conjugates linking irinotecan with TDO inhibitors increase the numbers of total CD4+/CD8+ T cells in a murine.
	Immunotherapy + Targeted Agents	PD-1 blockade + Peg-IFNα: PD-1 blockade relieves immunosuppression, while Peg-IFNα increases CD8+ T-cell infiltration, among other effects; Bavi + Pembro: Bavi enhances the immune-activating effect of Pembro	PD-1 blockade + Peg-IFNα demonstrates enhanced anti-tumor activity in research; Bavi + Pembro increases the response rate in HCC patients from 16% to 32%.
	Immunotherapy + Locoregional Therapies	TACE + immune adjuvants: TACE induces tumor ischemia and antigen release, while immune adjuvants activate anti-tumor immunity and enhance therapeutic efficacy; PD-1 blockade + RFA: PD-1 blockade suppresses metastases, while RFA releases tumor antigens and recruits cytotoxic T lymphocytes; PD-1 blockade + SBRT: PD-1 blockade restores T cell activity, while SBRT exposes tumor-specific antigens and amplifies immune response	Preclinical models show that shifting from total to partial embolization enhances efficacy and preserves liver function; PD-1 blockade + RFA enhances anti-tumor immunity and reduces recurrence in preclinical models; PD-1 blockade + SBRT in unresectable HCC achieved a median PFS of 7.4 months and an 18-month survival rate of 61.1%.

ACT, Adoptive Cell Therapy; AHR, Aryl Hydrocarbon Receptor; Atezo, Atezolizumab; Beva, Bevacizumab; Bavi, Bavituximab; CAR-T, Chimeric Antigen Receptor T cell; CTL, Cytotoxic T Lymphocyte; DC, Dendritic Cell; FOLFOX, Folinic Acid, Fluorouracil, and Oxaliplatin; HCC, Hepatocellular Carcinoma; ICI, Immune Checkpoint Inhibitor; Ipi, Ipilimumab; ORR, Objective Response Rate; Pembro, Pembrolizumab; Peg-IFNα, Pegylated Interferon Alpha; PD-1, Programmed Death-1; PD-L1, Programmed Death Ligand-1; PFS, Progression-Free Survival; RFA, Radiofrequency Ablation; SBRT, Stereotactic Body Radiation Therapy; TACE, Transcatheter Arterial Chemoembolization; TDO, Tryptophan 2,3-Dioxygenase; TICs, Tumor-Initiating Cells; VEGF, Vascular Endothelial Growth Factor.

### 4.3 Emerging therapeutic strategies for liver cancer TICs

Multiple therapeutic strategies are being developed to address the challenges posed by liver cancer TICs. The Wnt/β-catenin pathway is crucial in liver cancer, and 6-C-(E-phenylethenyl) naringenin (6-CEPN) inhibits TIC self-renewal, migration, and invasion by activating GSK3β, leading to the degradation and suppression of the Wnt/β-catenin pathway (52). In addition, selective tyrosine kinase inhibitors, such as Indo5, target the c-Met and Trk receptor tyrosine kinases, which are frequently co-activated in HCC and associated with poor prognosis (53). By blocking these pathways, Indo5 disrupts the related signaling and suppresses tumor growth. Equally important, Bio-SS-TS, a compound combining biotin and taraxasterol (TS), exploits the overexpression of biotin receptors on tumor cells for targeted delivery (54). It enhances mitochondria-dependent apoptosis and inhibits tumor development.

In parallel, genome editing also holds great promise in targeting liver cancer TICs. Marayati et al. used CRISPR/Cas9 to knockout PIM3 in hepatoblastoma cells, leading to reduced cell proliferation, viability, and stemness (55). Regarding non-coding RNAs, they are emerging as crucial therapeutic targets. Li et al. engineered an artificially designed lncRNA that functions as a molecular sponge, sequestering multiple oncogenic miRNAs and inhibiting tumor growth in xenograft HCC mouse models (56). Yao et al. demonstrated that miR-186 suppresses the expansion of liver CSCs by targeting PTPN11 (57). These advances suggest promising avenues for personalized TIC-targeted therapies.

### 4.4 Emerging therapeutic strategies for liver cancer TICs

Overcoming the liver’s immunosuppressive microenvironment is a key area for future research. Recent studies have identified several mechanisms by which TICs facilitate immune suppression and resistance (58). The overexpression of CD155 on TICs interacts with immune checkpoint receptors, leading to T cell exhaustion. Combining CD155 blockade with anti-PD-1/PD-L1 therapy has shown enhanced anti-tumor responses in preclinical models (12).

Additionally, a unique subset of TAMs expressing CD19 has been discovered in HCC. CD19+ TAMs exhibit immunosuppressive functions by upregulating PD-L1 and CD73, thereby inhibiting T cell activity, and direct targeting with anti-CD19 CAR-T cells has been shown to suppress tumor growth (59).

The immunosuppressive TME in HCC is further characterized by diverse immune cell populations and signaling pathways that impede effective immune responses (7). Strategies to overcome this include the use of inhibitors targeting the TGF-β/IL-10 pathway and metabolic modulators to enhance immune activation (60, 61). Moreover, accurately assessing and predicting tumor responses to immunotherapy remain critical challenges. Artificial intelligence (AI) integrating imaging, pathology, genomics, and clinical data show promise in predicting treatment responses and survival outcomes in HCC patients undergoing immunotherapy (62). Furthermore, MRI-based response criteria, particularly 3D quantitative enhancement tools (qEASL), have significant potential for predicting overall survival and identifying non-responders (63).

## 5 Conclusion

Liver cancer remains a major global health challenge, largely driven by TICs, characterized by robust self-renewal capabilities, plasticity, and immunosuppressive properties. These TICs actively remodel the TME employing mechanisms such as the overexpression of immune checkpoint molecules like CD155, recruitment of immunosuppressive macrophages including CD19-positive TAMs, and regulation of metabolic and cytokine pathways such as TGF-β and IL-10, collectively enabling immune evasion and sustained therapeutic resistance. Consequently, despite advances in immunotherapy, the efficacy of ICIs, adoptive cell therapies, and tumor vaccines is constrained by these sophisticated TIC-mediated immune escape mechanisms.

Emerging therapeutic strategies aim to integrate multimodal approaches that comprehensively disrupt TIC-driven immune suppression and resistance. Combination therapies merging immunotherapy with cytotoxic chemotherapy, molecular-targeted agents, and locoregional interventions such as TACE and RFA have shown clinical promise by enhancing antigen release, promoting immune cell infiltration, and reversing immunosuppressive metabolic states. Cutting-edge technologies, including genome editing via CRISPR/Cas9 for targeting TIC-specific pathways, nanotechnology-enhanced drug delivery systems, and novel cellular therapies directed against immunosuppressive cell populations, offer substantial promise for refining treatment efficacy. Furthermore, advanced predictive models leveraging AI and integrating multi-omics and imaging data may significantly enhance patient stratification and optimize immunotherapeutic outcomes.

However, several critical scientific challenges remain unresolved in the context of TIC-directed immunotherapy. These include the precise identification of TIC subpopulations most responsible for immune evasion, the dynamic evolution of their phenotypes under therapeutic and immune pressure, and the absence of reliable biomarkers to predict treatment responsiveness. Furthermore, the molecular mechanisms underlying the crosstalk between TICs and immunosuppressive stromal components are not yet fully elucidated. Addressing these gaps is essential for future progress in liver cancer therapy, which hinges on acquiring deeper mechanistic insights into TIC biology, immune resistance dynamics, and the complex interactions between TICs and their microenvironment. An improved understanding of these networks will provide a foundation for the development of durable and personalized immunotherapeutic strategies, ultimately aiming to enhance clinical efficacy and improve patient outcomes.
